# Washed microbiota transplantation via colonic transendoscopic enteral tube rescues severe acute pancreatitis: A case series

**DOI:** 10.1016/j.heliyon.2024.e33678

**Published:** 2024-06-26

**Authors:** Changjiang Huang, Cheng Huang, Renfu Tian, Yungang Pu, Pengfei Chen

**Affiliations:** a Intensive Care Unit, The Central Hospital of Enshi Tujia and Miao Autonomous Prefecture, Enshi, China; b Gastroenterology, The Central Hospital of Enshi Tujia and Miao Autonomous Prefecture, Enshi, China

**Keywords:** Fecal microbiota transplant, Case report, Pancreatitis, Infection, Transendoscopic enteral tubing

## Abstract

**Background:**

Gut microbiota dysbiosis plays a significant role in the development of acute pancreatitis (AP). However, a recent randomized trial reported negative findings regarding the use of fecal microbiota transplantation (FMT) via the mid-gut tube in severe AP. The case series presents the feasibility of washed microbiota transplantation (WMT) as a new methodology of FMT and its delivery via colonic transendoscopic enteral tubing (TET) for severe AP.

**Case series:**

We presented two cases of severe AP rapidly rescued using WMT via colonic TET. Symptoms related to severe AP and the acute physiology and chronic health evaluation-II score improved soon after WMT. In Case 1, bilirubin and infection indexes continuously decreased after the initial WMT and the patient was successfully weaned off the ventilator and recovered from multiple organ system failures (MSOF) within ten days. In Case 2, the patient's consciousness rapidly improved within one day after WMT, with normal bowel sounds and stable blood pressure without vasoactive drug maintenance. Both Case 1 and Case 2 completed follow-ups of seven months and twenty-two months, respectively, with no reports of new-onset diabetes.

**Conclusion:**

WMT via colonic TET played a critical therapeutic role in rescuing severe AP cases. This is the first report providing direct evidence for the clinical value of targeting microbiota through colonic TET in rescuing severe AP.

## 1 Introduction

Acute pancreatitis (AP) is an inflammatory injury caused by various etiological factors leading to self-digestion of the pancreatic tissue. Severe AP is defined by the presence of organ failure, which significantly escalates the mortality rate among affected patients [1]. In addition to short-term disease burden, 25 % of patients with necrosis pancreatitis would develop new-onset diabetes within the first year [2]. Recently, increasing studies demonstrated that gut microbiota played a pivotal role in the development of AP [3,4]. Gut microbiota dysbiosis in patients with AP could be characterized as gut microbial diversity significantly decreased [5]. Besides, therapeutic interventions targeting gut microbiota were also considered as a new front in AP treatment [6].

However, a randomized trial using fecal microbiota transplantation (FMT) for severe AP didn't observe the difference in intra-abdominal pressure [7]. This study delivered FMT through the nasojejunal tube for the FMT group (n = 30) and saline for the control group (n = 30). The negative findings inspired us to explore new methods of using FMT for AP, such as different delivery ways, doses, frequency, and timing for transplants.

The colon is the core place where gut microbiota interacts with the host. Considering the severe intestinal dysfunction and mucosal barrier damage in severe AP patients, the functional microbiota might not be safely transmitted to the colon in time through the mid-gut. The colonic transendoscopic enteral tubing (TET), a regular technique for rescuing endoscopy-associated perforation in some therapeutic centers by drainage air and fluid [8], should be a new option for FMT delivery in these AP patients. The improved methodology of FMT based on automatic washing process and related delivering consideration, named washed microbiota transplantation (WMT), changed the FMT-related safety, quantitative method, and delivery of microbiota suspension [9]. Furthermore, WMT showed reduced microbiota burden and inflammation burden in the transplants compared with manual FMT [10]. Considering that WMT is a non-profit medical technology permitted in China [11], and moreover, colon TET tube is a routine marketing available endoscopic device in China [12], WMT via colonic TET could provide a more reasonable treatment modality for patients with severe AP in practice. Here, we present our findings on the short-term efficacy and long-term follow-up of WMT via colonic TET for severe AP.

## 2 Case series presentation

### 2.1 Case 1

**Fig. 1:**
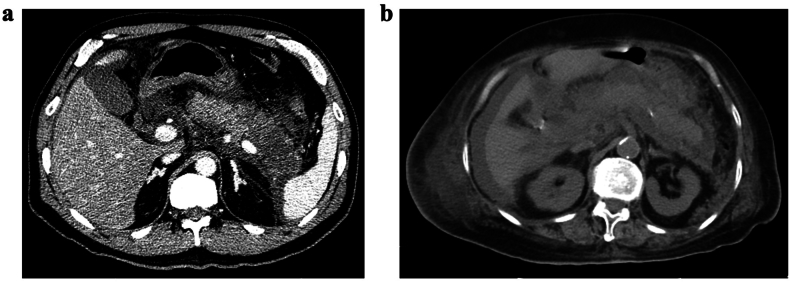
Abdominal CT showed SAP before the starting of WMT (a: Case 1; b: Case 2).

**Fig. 2:**
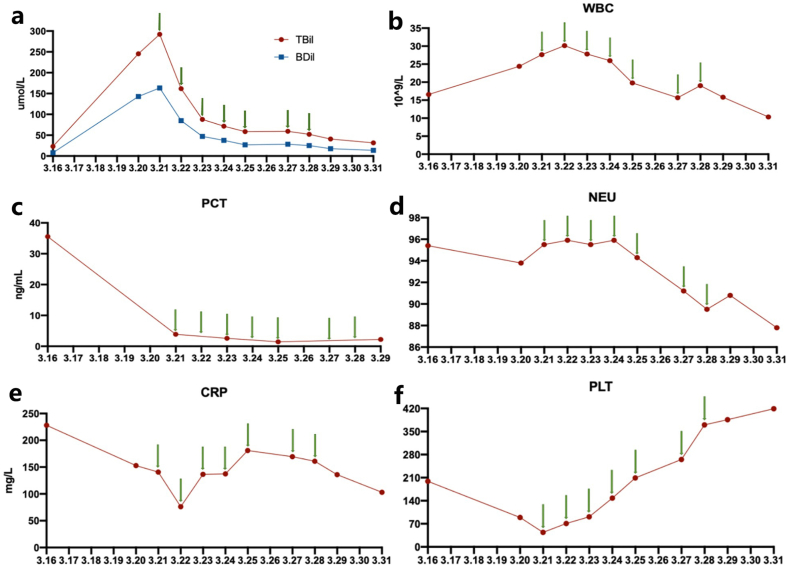
Trends of bilirubin and key inflammatory indicators in the Case 1 before and after WMT treatment. The green arrow represents WMT intervention. a: TBiL: total bilirubin; BDiL: direct bilirubin. b: WBC: white blood count. c: PCT: procalcitonin. d: NEU: the percent of neutrophilic granulocyte. e: CRP: C-reactive protein. f: PLT: blood platelet. (For interpretation of the references to colour in this figure legend, the reader is referred to the Web version of this article.)

A 59-year-old male was referred to hospital due to persistent upper abdominal pain and nausea on March 14, 2022. On admission, CT confirmed the diagnosis of acute necrotizing pancreatitis (Table 1, Fig. 1a). He had a history of hyperlipidemia and had stopped using hypolipidemic drugs for 2 months. His condition deteriorated two days later and was transferred to the intensive care unit (ICU). He developed intra-abdominal hypertension (bladder pressure: 26 cmH_2_O), with hypoactive bowel sound, shortness of breath, reduced blood pressure, and oxygen saturation. He was diagnosed with severe AP, acute respiratory distress syndrome (ARDS), multiple system organ failure (MSOF), pulmonary infection, metabolic acidosis, hyperlipidemia, and septic shock. The acute physiology and chronic health evaluation-II (APACHE-II) score was 24. Comprehensive treatment was performed with less improvement. On March 18, the patient developed pancreatic encephalopathy, and respiratory distress worsened requiring endotracheal intubation and ventilation. After multidisciplinary consultation, the panel concluded that WMT may provide a rescue value for him. From March 21 to 28, he underwent 7 WMTs (1 unit per delivery, a total of 7 units) through colonic TET. His condition gradually improved as expected. The levels of bilirubin and infection markers rapidly decreased (Fig. 2), and the intra-abdominal pressure declined to normal after WMTs. Two days after the initial WMT, a physical examination indicated stable vital signs, soft abdomen, and resuming bowel sound, then the vasoactive drugs were discontinued, and the endotracheal intubation and ventilation were taken off. Six days after the initial WMT, nasojejunal feeding was given (Supplementary Table 1). He was transferred out of the ICU with an APACHE-II score of 5 on April 1. Our physicians suggested conservative treatment and puncture drainage, however, the patient's family insisted on transferring to another hospital for surgical intervention. Eight days later, he underwent a partial pancreatectomy and jejunostomy, and he was discharged smoothly following the surgery. During the follow-up period, the patient did not report new-onset diabetes. No WMT-related adverse events were observed. However, after seven months, he was admitted to the emergency department due to small intestinal obstruction. No pancreatic abnormality was detected during the comprehensive examination. The patient's condition did not improve, and he died four days after the admission.

**Table 1:** Laboratory findings of the patients on admission to the ICU.

Testing items		Results		Reference range
		Case 1	Case 2	
Blood Routine Examination	WBC, x 10^9^/L	24.59	17.88	4–10
	NEU, %	94.9	98.3	50–70
	PLT, x 10^9^/L	370	200	90–300
	CRP, mg/L	228.03	177.34	0–6
	Hb, g/L	177	126	110–150
–	PCT, ng/ml	35.54	23.73	0–0.5
Blood biochemical test	TP, g/L	66.13	53.26	60–82
	Albumin, g/L	41.69	30.44	35–53
	ALT, U/L	65	16	5–40
	AST, U/L	123	31	5–40
	Scr, umol/L	123.1	8.5	35–110
	TC, mmol/L	12.83	1.5	2.8–5.8
	TG, mmol/L	16.55	1.12	0.4–1.53
	serum amylase, IU/L	1831.00	138	34–97
Arterial blood gas	PH	7.14	7.15	7.35–7.45
	PCO_2_, mmHg	35.0	57.32	35–45
	PO_2_, mmHg	75.0	63.11	80–100
	Glucose, mmol/L	11.20	8.07	4.1–5.9
	Ca^2+^, mmol/L	0.6	1.28	1.12–1.32

WBC, white blood count; NEU%, the percent of neutrophilic granulocyte; PLT, blood platelet; CRP, C-reactive protein; Hb, hemoglobin; (PLT); PCT, procalcitonin; TP, total protein; ALT, alanine aminotransferase; AST, aspartate transaminase; Scr, serum creatinine; TC, total cholesterol; TG, triglyceride; PCO_2_, partial pressure of carbon dioxide; PO_2_, partial pressure of oxygen.

### 2.2 Case 2

An 86-year-old female was admitted to hospital with epigastric pain on April 2, 2022. The serum amylase (2145.00 U/L) was remarkably elevated. The CT scan and abdomen ultrasound showed AP with intrahepatic bile ductal stones in the common bile duct, and pancreatic ductal dilatation, cholangiolithiasis (Fig. 1b). She had undergone coronary stenting, left atrial appendage occlusion, and laparoscopic cholecystectomy. Her condition worsened with ARDS, MSOF, shock, atrial fibrillation, and pulmonary infection on day 13 after admission. She was transferred to the ICU with an APACHE-II score of 25 (Table 1). Although comprehensive treatment was given, her condition worsened with the disappearance of bowel sounds. She underwent repeated WMTs (1 unit per delivery, a total of 7 units) by colonic TET from April 24 to 29. Her consciousness improved rapidly within one day after the initial WMT, with normal bowel sound and stable blood pressure without vasoactive drug maintenance (Supplementary Table 2). The CT on April 27 showed a reduction of pancreatic exudation, bilateral abdominal wall exudation, and peritoneal effusion. Her APACHE-II score decreased to 9 on May 2. In subsequent assessments, her bilirubin levels continued to decrease, and inflammatory markers also exhibited a declining trend (Table 2). Moreover, her vital signs remained stable, and she was successfully discharged approximately one month after the initial WMT. Throughout the 22-week follow-up period, the patient's overall condition remained favorable, with no development of new-onset diabetes, intestinal obstruction, or other complications, and no additional surgical interventions were deemed necessary. No WMT-related adverse events were observed.

**Table 2:** Trends of bilirubin and key inflammatory indicators in Case 2 before and after WMT treatment.

Date	TBil (2-20μmol/L)	BDil (0–6 μmol/L)	PCT (0–0.5ng/mL)	WBC (4–10 × 109/L)	NEU% (50–70)	PLT (90–300 × 109/L)	CRP (0–6mg/L)
4.23	94.7	41.4	2.27	9.87	82.9	41	110.5
4.25, the 2nd WMT	104.7	48.3	3.24	7.95	84.6	60	42.81
4.27, the 4th WMT	99.7	41	3.1	10.32	88.5	62	40.5
4.29, the 6th WMT	46.6	22.3	3.72	13.31	89.1	145	43.1
5.2	46	15.3	7.42	13.08	82.4	214	77.57
5.16	27.9	10.5	0.4	8.76	77.00	190	60.50
5.21	23.7	7.4	0.41	6.05	68	206	50.50

TBiL: total bilirubin; BDiL: direct bilirubin; WBC: white blood count; PCT: procalcitonin; NEU: the percent of neutrophilic granulocyte; CRP: C-reactive protein; PLT: blood platelet.

## 3 Discussion

Given the difficulty in bowel preparation for these patients, severe pancreatitis patients initially have little to no food intake. Both cases had nasogastric tubes and received treatment with Rhubarb herbal liquid via nasal feeding and enema. Enemas can be used for bowel preparation. During the placement of the colonic TET tube in severe pancreatitis patients, it is essential to ensure hemodynamic stability. For patients in shock, vasoactive drugs should be used, and for those with pancreatic encephalopathy, appropriate sedatives, and analgesics should be administered. This procedure can be performed with the cooperation of the anesthesiology department. A gastroenterologist then performed a bedside colonoscopy and placed the colonic TET tube into the cecum, securing the TET tube with titanium clips.

In the present study, both cases benefited from WMT, leading to rapid improvement in inflammation and MSOF. Admission with Ca^2+^ levels below 1.94 mmol/L and pH below 7.37 has been identified as predictive of elevated fatality rates in severe AP [13]. The lower pH and Ca2+ levels observed in our current cases indicated a poor prognosis. Despite receiving timely and comprehensive treatments, both cases experienced worsening MSOF, shock, and peritonitis. Furthermore, the progression of AP and consistent medication usage might exacerbate dysbiosis in the gut microbiota, consequently worsening the severity of AP [14]. A recent study suggested that gut microbiota composition might be a superior predictor of AP severity compared to established severity scores [3]. Therefore, reconstructing the gut microbiota became paramount to breaking this detrimental cycle. Conversely, a previous study in mice reported that antibiotics decreased mortality in necrotizing pancreatitis mice on a Western-type diet, while FMT increased mortality and bacterial dissemination [15]. Additionally, a clinical study failed to observe improvement in AP after FMT via nasojejunal tube [7]. These disparate findings compared to our study might serve as pivotal points for advancing microbiota reconstruction in AP treatment.

Previous systematic review revealed that the severe acute inflammation in small intestine, the indication of the mucosal barrier injury, is a risk factor of FMT-related serious adverse events [16]. AP has been shown to increase intestinal permeability [17], potentially leading to bacterial translocation from the intestines to the pancreas, thereby perpetuating inflammation. Given the close anatomical relationship between the pancreas and the small intestine, such as through the communication of the pancreaticobiliary duct with the duodenum, microbiota transferred through the small intestine in AP might have negative consequences, as seen in previous clinical and mice studies [7,15]. Additionally, AP patients often experience intestinal failure as part of MSOF, resulting in weakened intestinal motility [18]. This prolonged retention of transplanted microbiota in the small intestine further elevates the risk of bacterial translocation. In the present study, both patients underwent WMT via colonic TET, thereby avoiding the aforementioned risks associated with introducing microbiota burden into the more permeable and less motile small intestine. As a new generation FMT technology, WMT has demonstrated remarkable clinical convenience, safety, and therapeutic efficacy [11]. Colonic TET is a procedure that entails the insertion and fixation of a small and flexible TET tube into the colon under endoscopic guidance [19]. The location of the fixation of the TET tube is flexible, depending on the clinical requirements and intestinal condition [12]. It has been proven effective and safe in various clinical scenarios, including the treatment of perforations [8,20], bridging emergent intestinal obstructions to scheduled surgery [21], and collecting colonic samples for microbiome research [22,23]. Microbiota medicine, as an emerging clinical discipline, has introduced colonic TET as a novel therapeutic approach, bridging microbiome research with diverse academic disciplines [24].

In the present study, we extended the application of colonic TET as a delivery route for WMT in the treatment of severe AP. The rapid improvement of patients' conditions following the initial WMT without any reported adverse events underscored the efficacy and safety of WMT via colonic TET in AP. In Case 1, bilirubin levels and infection indexes steadily decreased after the initial WMT, facilitating ventilator removal and recovery from MSOF within a mere 7 days of WMT initiation from onset. This outcome provided valuable evidence regarding the timing of WMT administration in serious diseases. In Case 2, WMT partially ameliorated severe AP; however, advanced age, multiple severe comorbidities, and unaddressed biliary stones prolonged hospitalization compared to Case 1. Notably, antibiotics were continued during WMT, reflecting the adoption of a step-up FMT strategy, which involves combining narrow-spectrum antibiotics with WMT for severe infections [25]. Sequential FMTs with interval antibiotics have shown promise in improving outcomes in patients with severe or severe-complicated *Clostridioides difficile* infection [26].

Although both cases showed improvement in severe AP during hospitalization, the long-term burden, including disease recurrence, the need for surgery, and pancreatic insufficiency, remains significant [27]. In the present study, Case 1 underwent surgery shortly after pancreatitis improvement during hospitalization. However, this procedure might have increased the risk of postoperative intra-abdominal adhesions, ultimately leading to death due to small intestinal obstruction seven months later. Despite being an elderly patient with more severe underlying medical conditions, Case 2 did not require surgery during the nearly two-year follow-up period. Additionally, neither case developed new-onset diabetes during our follow-up. The reported morbidity rates of new-onset diabetes in patients with necrotizing pancreatitis were 25 % over one year [2], and 43 % over 24 months [28]. WMT might have contributed to this outcome, as gut microbiota likely plays a crucial role in preventing new-onset diabetes following AP [29]. There are limitations in this case series. Integrative analyses involving microbial, metabolic, and immunologic detection could facilitate future mechanistic research.

## 4 Conclusion

We report the experiences based on two consecutive cases of successful treatment of severe AP using WMT via colonic TET. WMT via colonic TET offers a reasonable, safe, and effective strategy for clinicians to manage severe AP. The findings from this study offer novel insights into the clinical significance of targeting the microbiota in AP treatment.

## Ethical statement

This study was approved by the Institutional Review Board of the Central Hospital of Enshi Tujia and Miao Autonomous Prefecture and the non-profit WMT sourcing institution of the Second Affiliated Hospital of Nanjing Medical University ([2022]-KY-057-01). Written informed consent was obtained from patients and their legal guardians. All procedures were followed by the Helsinki Declaration of 1975.

## Data availability statement

Data will be made available on request.

## Funding

None.

## CRediT authorship contribution statement

**Changjiang Huang:** Writing – review & editing, Writing – original draft, Resources, Data curation, Conceptualization. **Cheng Huang:** Writing – review & editing, Investigation, Data curation. **Renfu Tian:** Writing – review & editing, Investigation. **Yungang Pu:** Writing – review & editing. **Pengfei Chen:** Writing – review & editing, Supervision, Methodology.

## Declaration of competing interest

The authors declare that they have no known competing financial interests or personal relationships that could have appeared to influence the work reported in this paper.

## Abbreviations

AP: acute pancreatitis
FMT: fecal microbiota transplantation
WMT: washed microbiota transplantation
TET: transendoscopic enteral tubing
MSOF: multiple organ system failures
ICU: intensive care unit
ARDS: acute respiratory distress syndrome
APACHE-II: acute physiology and chronic health evaluation-II
