# Supplementary material for: Washed microbiota transplantation via colonic transendoscopic enteral tube rescues severe acute pancreatitis: A case series
Source: Heliyon. 2024 Jun 26;10(13):e33678. doi: 10.1016/j.heliyon.2024.e33678 (PMC11279248; doi:10.1016/j.heliyon.2024.e33678)
Supplement: Multimedia component 1 [file mmc1.docx]

| Supplementary Table 1 Case 1: Medication, blood purification, and ventilator usage before and after WMT | | | | |
| --- | --- | --- | --- | --- |
| Date | Antibiotic | Vasopressor | Ventilation and Blood Purification | Enteral Nutrition |
| Day 2 | Piperacillin-tazobactam | Norepinephrine | Non-invasive ventilator, Plasma exchange + CRRT | Fasting |
| Day 6 | Meropenem | Norepinephrine | Invasive ventilator P-SIMV mode, CRRT | Fasting |
| Day 7 (1st WMT) | Meropenem | Norepinephrine | Invasive ventilator, CRRT | Fasting |
| Day 8 (2nd WMT) | Meropenem | Norepinephrine | Invasive ventilator, CRRT | Fasting |
| Day 9 (3rd WMT) | Meropenem | Norepinephrine | Discontinued ventilator | Fasting |
| Day 10 (4th WMT) | Meropenem | Discontinued norepinephrine | Discontinued ventilator | Fasting |
| Day 11 (5th WMT) | Meropenem | No norepinephrine | No ventilator | Glucose injection nasal feeding |
| Day 13 (6th WMT) | Meropenem | No norepinephrine | No ventilator | Enteral nutrition |
| Day 14 (7th WMT) | Meropenem | No norepinephrine | No ventilator | Enteral nutrition |
| Day 15 | Meropenem | No norepinephrine | No ventilator | Enteral nutrition |
| Day 17 | Piperacillin-tazobactam | No norepinephrine | No ventilator | Enteral nutrition |

CRRT, Continuous Renal Replacement Therapy; WMT, Washed Microbiota Transplantation
