# Supplementary material for: Washed microbiota transplantation via colonic transendoscopic enteral tube rescues severe acute pancreatitis: A case series
Source: Heliyon. 2024 Jun 26;10(13):e33678. doi: 10.1016/j.heliyon.2024.e33678 (PMC11279248; doi:10.1016/j.heliyon.2024.e33678)
Supplement: Multimedia component 2 [file mmc2.docx]

| Supplementary Table 2 Case 2: Medication, blood purification, and ventilator usage before and after WMT | | | | |
| --- | --- | --- | --- | --- |
| Date | Antibiotic | Vasopressor Usage | Ventilation and Blood Purification | Enteral Nutrition |
| Day 15 | Meropenem + Tigecycline | Norepinephrine | Non-invasive ventilator and CRRT | Fasting |
| Day 21 | Meropenem | Norepinephrine | None | Fasting |
| Day 22 (1st WMT) | Meropenem | Discontinued norepinephrine | None | Enteral nutrition |
| Day 23 (2nd WMT) | Meropenem | None | None | Enteral nutrition |
| Day 24 (3rd WMT) | Cefoperazone-sulbactam | None | None | Enteral nutrition |
| Day 25 (4th WMT) | Cefoperazone-sulbactam + Moxifloxacin | None | None | Enteral nutrition |
| Day 26 (5th WMT) | Cefoperazone-sulbactam + Moxifloxacin | None | None | Enteral nutrition |
| Day 27 (6th WMT) | Meropenem + Tigecycline | None | None | Enteral nutrition |
| Day 30 | Meropenem + Tigecycline | None | None | Enteral nutrition |

CRRT, Continuous Renal Replacement Therapy; WMT, Washed Microbiota Transplantation.
